# Ghrelin, not corticosterone, is associated with transitioning of phenotypic states in a migratory Galliform

**DOI:** 10.3389/fendo.2022.1058298

**Published:** 2023-01-09

**Authors:** Valeria Marasco, Hiroyuki Kaiya, Gianni Pola, Leonida Fusani

**Affiliations:** ^1^ Konrad Lorenz Institute of Ethology, University of Veterinary Medicine, Vienna, Vienna, Austria; ^2^ Department of Biochemistry, National Cerebral and Cardiovascular Center Research Institute, Suita, Japan; ^3^ Research Division of Drug Discovery, Grandsoul Research Institute for Immunology, Inc., Nara, Japan; ^4^ Istituto Sperimentale Zootecnico per la Sicilia, Palermo, Italy; ^5^ Department of Behavioural and Cognitive Biology, University Biology Building, University of Vienna, Vienna, Austria

**Keywords:** phenotypic flexibility, avian migration, migratory state, migratory fuelling, ghrelin, corticosterone

## Abstract

In both captive and free-living birds, the emergence of the migratory phenotype is signalled by rapid and marked increases in food intake and fuelling, as well as changes in amount of nocturnality or migratory restlessness. The metabolic hormone corticosterone and, as more recently suggested, the gut-derived hormone ghrelin have been suggested to play a role in mediating such phenomenal phenotypic flexibility given that they both regulate fuel metabolism and locomotion across vertebrate taxa. Here, using the Common quail (*Coturnix coturnix*) as our study species, we induced autumn migration followed by a non-migratory wintering phase through controlled changes in daylight. We thus compared plasma corticosterone and ghrelin concentrations between the two sampling phases and assessed whether these hormones might reflect the migratory state. While we found no differences in plasma corticosterone between the two sampling phases and no link of this hormone with changes in body mass, levels of food intake or migratory restlessness, the migratory birds had substantially higher levels of plasma ghrelin relative to the non-migratory birds. Furthermore, while ghrelin did not correlate with the gain in body mass over the entire pre-migratory fuelling phase (over an average of nine weeks preceding blood sampling), plasma ghrelin did positively correlate with the gain in body mass observed during the final fattening stages (over an average of three weeks preceding blood sampling). Again, variation in plasma ghrelin also reflected the amount of body mass depleted over both the long- and short-time frame as birds returned to their non-migratory baseline - lower levels of plasma ghrelin consistently correlated with larger losses in body mass. Thus, while our data do not highlight a role of the hormone corticosterone in sustaining pre-migratory fattening as shown in other bird species, they do add evidence for a potential role of ghrelin in mediating migratory behaviour and further suggest that this hormone might be important in regulating the transitioning of migratory states, possibly by promoting fuel mobilisation and usage.

## 1 Introduction

Most, if not all, migratory birds evolved spectacular physiological adaptations to accommodate their long-distance migratory flights between their breeding and wintering grounds reviewed in (1, 2). Extensive work has demonstrated that migratory birds use fat as the prime energy source for their migratory flights due to its higher efficiency compared to carbohydrates or proteins (3–6), reviewed by (6). The accumulation of energy reserves that many birds exhibit in preparation to their migratory flights – termed pre-migratory fuelling – is promoted by increased food intake (hyperphagia) which, subsequently, leads to rapid and remarkable gains in subcutaneous fat stores (6–9). Studies in different bird species sampled at stopover sites (i.e., where migrating birds temporarily suspend the migratory flight to rest and refuel) show that individuals with larger subcutaneous fat stores exhibit higher levels of migratory restlessness in captivity (10–14) – i.e., the urge of captive birds to migrate at night (14) and can migrate faster (13). Thus, the physiological preparations occurring over the phase of pre-migratory fuelling are likely to be central in determining the success of the subsequent active phases of the migratory cycle. While it is relatively well known that the motivation of migratory birds to fuel is controlled by changes in photoperiod (2, 15) and by endogenous programs (16–18), the exact endocrine regulators of pre-migratory fuelling remain little understood.

In vertebrates, glucocorticoid hormones (corticosterone is the primary avian glucocorticoid, while it is cortisol in most mammals and fish) play a permissive role in feeding behaviour, energy mobilisation, as well as locomotion (19–22). Thus, it was originally hypothesized that corticosterone plays roles in stimulating fattening and promoting physiological preparation to migration [reviews (23, 24)]. Various studies across different bird species have tested this hypothesis either by experimentally manipulating corticosterone signalling and examining its effect on measures of feeding behaviour and changes in body mass/fat stores e.g (25–28), or by assessing the link between naturally circulating levels of corticosterone and measures of physiological preparations in birds transitioning into the migratory state or at stopover sites e.g (29–33). As highlighted by the recent review of Bauer and Watts (24), these studies provided very limited support that corticosterone promote pre-migratory fuelling (i.e. *Physiological Preparation Hypothesis*). In fact, despite it is often the case that corticosterone levels are elevated in birds that had reached their peak of body mass (26, 29, 30, 34), changes in plasma corticosterone most often do not parallel with changes in body mass review (24). Thus, corticosterone might sustain enhanced body condition and maintain peak levels of fat deposits (30, 35), and/or have a role in stimulating or maintaining increased activity levels during the active migratory flights (*Departure Stimulation Hypothesis, or the Flight Support Hypothesis, respectively* – (24)), though fewer studies have tested the latter hypotheses.

Another endocrine candidate that has started to receive a growing attention in the field of avian migration is the recently discovered gut-derived appetite-regulating hormone ghrelin (36). Ghrelin is highly conserved in vertebrates (36) and occurs in two distinct isoforms - acylated and unacylated ghrelin (37, 38). Unacylated ghrelin was originally considered an inactive form of ghrelin because it does not bind to the growth hormone secretagogue receptor (GHSR-1a). Although the regulation of the ghrelin axis remains to be elucidated, it is now clear that unacylated ghrelin exerts a number of systemic functions likely by binding with alternative ghrelin receptors that remain to be identified [reviewed in (39)]. Knowledge about effects of ghrelin on energy metabolism comes mostly from studies in mammals (humans and rodents) and poultry (domestic chickens and Japanese quails). The effect of ghrelin on food intake and lipid metabolism appear to differ between these two taxa as in humans and rodents ghrelin promotes fat storage and feeding (40–42), while in chickens and quails it inhibits them (43–45). Furthermore, exogenous ghrelin leads to corticosterone release in chickens (46–48), suggesting that ghrelin signalling may be functionally linked with glucocorticoids. Recent studies on migratory passerines caught at stopover sites during spring migration suggested that ghrelin can influence stopover and departure decisions (49, 50), though mechanisms might differ during autumn migration (51). In free-flying garden warblers (*Sylvia borin*) it was first shown that migrants caught at the stopover site with completely depleted fat stores had on average lower plasma concentrations of acylated ghrelin (the only isoform that can currently be measured in avian plasma) than the birds still carrying fat stores (49). It was also shown, importantly, that the exogenous administration of ghrelin in birds that were temporarily caged altered migratory restlessness and food intake, but effects depended on the injected isoform and the birds’ nutritional conditions (49). In a follow-up study on a different passerine species – the yellow-rumped warbler (*Setophaga coronata coronata*)–Lupi et al. (50) showed that the experimental increase of both ghrelin isoforms in free-flying migrants caused a rapid and short-term acceleration of departure from the stopover site. Overall, these data suggested that ghrelin might promote migratory movements (50) possibly through the stimulatory effects of this hormone on fat breakdown (46). However, whether ghrelin is functionally linked with physiological adaptations during pre-migratory fuelling is currently unknown as all research so far was conducted in active migrants that had already undergone this phase and varied largely in their nutritional conditions at sampling (stopover).

Here, we experimentally controlled the migratory state of young adult Common quails (*Coturnix coturnix*) by simulating autumn migration followed by a non-migratory phase. The common quail is the only migratory Galliform of the western Palearctic (52). The species breeding area is broadly distributed in Eurasia, while the overwintering distribution ranges from sub-Saharan to Central/North Africa to southern India (53). Wild common quails show large variability in their migratory routes and distances with populations wintering south of the Sahara migrating longer distances compared to populations wintering north of the Sahara (between 25-35 N°). Common quails show physiological adaptations to migration similar to those found in most Passerines species, including remarkable increases in nocturnal activity and fat deposition at the onset of the migratory season in captive conditions (54–56), and the temporal interruption of their migratory flights at stopover sites (57). Furthermore, the extensive knowledge on photoperiodic control system, husbandry and breeding in captivity of the closely related Japanese quail - *Coturnix japonica* (58) makes the Common quail a good laboratory model for studying migratory physiology, in particular to examine the endocrine changes associated with pre-migratory fuelling while controlling for potential confounding factors such as variation in age, sex, and circadian rhythmicity of experimental animals which would be very difficult to achieve in nature. In this study, we compared plasma concentrations of corticosterone and ghrelin between quails sampled during the migratory phase (i.e. when they reached their peak of body mass), or the non-migratory phase (i.e., as they returned to their physiological baseline). We thus assessed whether these hormones reflected the migratory state of the birds estimated through measurements of food intake, migratory restlessness, and changes in body mass. As corticosterone might have a role in maintaining peak levels of fuel in migratory birds ready to depart (30, 35), we predicted that plasma corticosterone would be higher in the birds sampled during the migratory phase than the non-migratory phase. It is difficult to make exact predictions about the relationships between migratory state and ghrelin given the limited research so far. However, the use of a migratory Galliform as our study system is particularly advantageous in that it allowed us to cross check function of ghrelin known for the few studied migratory Passerines and the domestic Galliformes. Given the inhibitory effects of ghrelin on food intake and its stimulatory effect on lipid breakdown in the domestic Galliformes (citations above), we predicted that plasma ghrelin would reflect changes in body mass as birds were transitioning to a migratory, or non-migratory state. Furthermore, as the hormonal measurements were performed within the same individual birds we also tested whether circulating levels of ghrelin were positively correlated with measures of corticosterone.

## 2 Materials and methods

### 2.1 Study subjects and housing conditions

This study was performed on a subset of subjects used for a larger experiment published elsewhere (full details on experimental design in (56). Briefly, eggs of Common quail were obtained from our breeding stock kept at the Konrad Lorenz Institute of Ethology (Vetmeduni Vienna, Austria). The stock quails originated from wild funders captured along the coast of Palermo (Sicily, Italy) that showed no signs of admixture with Japanese quails (59). The eggs were artificially incubated (incubator: MG 70/100 F, FIEM srl, Italia) and hatchlings reared under a 16:8 hrs light:dark cycle until they were 7 weeks of age. When they could be sexed (4 weeks of age), the birds were housed in sex-mixed groups of 11-12 in each enclosure (6 enclosures in total). Food (turkey starter, Lagerhaus, Austria) and water were always provided *ad libitum* throughout the course of the experiment. All birds were housed within a single room and the temperature was always maintained between 20-24°C. The experiment was performed in compliance with the Austria legislation with approval of the Ethics Committee of the University of Veterinary Medicine Vienna, and the Federal Ministry of Science, Research and Economy (BMWFW-68.205/0037-WF/V/3b/2017).

### 2.2 Photoperiod manipulation and assessment of physiological migratory state

**Figure 1:**
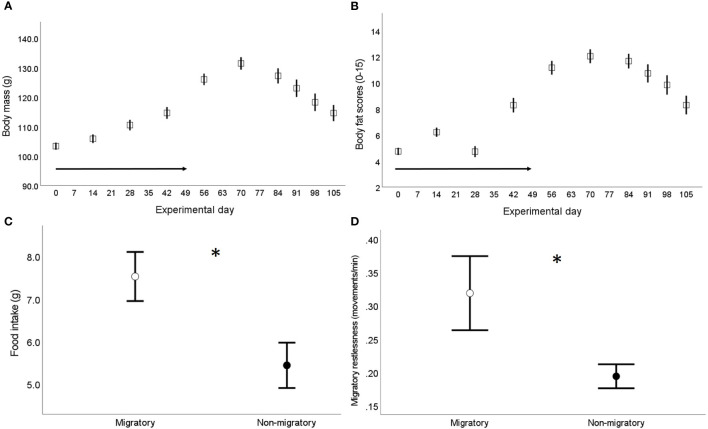
Assessment of physiological migratory state in the captive population of common quails used in the main experiment (56) – birds aged seven weeks old when the experiment started (experimental day 0). **(A)** Changes in body mass and **(B)** total subcutaneous fat scores (assessed in the furcular, scapular and abdomen following Boswell et al. (55) over the experimental period; the arrow in both figures indicates the weekly reduction in daylight (experimental days 0-49); sample size from experimental days 0-56: 68 birds, sample size from experimental days 70-105: 35 birds. **(C)** Food intake (g) and **(D)** migratory restlessness (i.e. average nocturnal activity levels) separately by sampling group (n=33 migratory and 35 non-migratory) assessed when the birds were singly housed, 1-3 days prior blood sampling in the migratory group (experimental days 56-70) and the non-migratory group (experimental days 105-119). Data are presented as means ± se; * indicates *p* < 0.05.

At seven weeks of age (indicated as experimental day 0), all birds part of the main experiment (n=68) were exposed to a gradual reduction of day length (30 min/week) until the photoperiod reached 12:12 hrs light:dark (experimental day 49) and was maintained constant until the end of the experiment. This light:dark schedule simulated autumn migration to (sub)tropical regions followed by a sedentary stage at the wintering grounds. As reported elsewhere (56), we determined the migratory state of the birds by regularly monitoring changes in body mass and subcutaneous fat scores over the entire experimental period. We found that quails increased their body mass (on average 22%) due to accumulation of body fat stores as the days were getting shorter (experimental days 0-56); while the birds rapidly lost body mass (on average 13%) due to a depletion of fat stores when the photoperiod was maintained constant – experimental days 70-105 (**Figures 1** **A, B** and **Table S1** in Supplementary Material). We further assessed the migratory state of the birds 1-3 days prior blood sampling (when birds housed within a same enclosure where singly housed) *via* measurements of food intake and nocturnal activity levels or migratory restlessness (full methodological details in **Supplementary Material**). Briefly, food intake was estimated in each individually housed bird by measuring the amount of food eaten from the start of the single housing (10:00-11:00 h) until the following 24 hours; while migratory restlessness was estimated by an infrared sensor system connected with an activity recorder that registered locomotor activity within each cage at 1-min intervals. As expected, migratory birds showed higher levels of food intake and migratory restlessness compared the non-migratory birds (**Figures 1** **C, D**, **Supplementary Material** and **Table S2**).

### 2.3 Blood sampling for measurements of plasma ghrelin and plasma corticosterone

Upon experimental day 56, 33 out of 68 birds were randomly chosen and allocated to the migratory sampling phase (experimental days 56-70); of this, 16 birds were sampled at 9:00 h (three hours after lights on), and 17 were sampled at 21:00 h (three hours after lights off). The remaining birds were allocated to the non-migratory sampling phase (experimental days 105-119); of this, 18 were sampled at 9:00 h and 17 were sampled at 21:00 h. We performed the hormonal measurements only on the subsets of birds sampled at 9:00h; samples collected at 21:00 h were meant to be used for other purposes (to be published elsewhere). Within each sampling phase, blood samples were taken over a consecutive period of twelve days in singly housed birds. We took blood samples (up to 400 µl) from a maximum of two birds in each sampling day to collect samples as close to the defined collection time as possible to control for variation due to circadian rhythms (60, 61) and to ensure reliable estimation of baseline physiological measurements (62). Thus, we collected blood samples within 2 min from entering the experimental room (mean ± se: 61.97 ± 1.38 sec, n=34; as expected, bleed time was not correlated with either corticosterone or ghrelin, -0.29 < Pearson’s r > 0.15, *p* ≥ 0.1) and immediately placed them on ice. Within 30 min from collection, blood samples were centrifuged to separate plasma from red blood cells. For each bird, we made two separate plasma aliquots; one aliquot (70-100 µl) was acidified using 1M HCL (dilution 1:10) before storing the samples at -80C for stabilization of acylated ghrelin in plasma as described in Goymann et al. (49).

### 2.4 Hormonal analyses

Corticosterone was extracted two times in 1 ml of diethyl ether (Carl Roth, Austria) from plasma aliquots (~20 μl). After extraction, corticosterone concentrations (ng/ml) were measured using an enzyme immunoassay following the manufacturer instructions (Assay Designs Corticosterone Kit 901-097, Enzo Life Sciences). Samples were run in two plates (equally spread in relation to sampling group) and the average intra-assay and inter-assay variation calculated using a plasma quail pool run in both plates were 6.5% and 11.1%, respectively; the detection limit of the assay was 0.2 ng/ml.

Plasma ghrelin levels were measured using a radioimmunoassay (RIA) that has been described previously (63, 64). Acidified plasma was validated to use directly for the RIA by serial dilution of the plasma without extraction similar to tilapia (65). A primary antibody that recognizes the N-terminal portion of octanoylated rat ghrelin (Gly1–Arg11) was used at a final dilution of 1/5,000,000. Octanoylated chicken ghrelin (chicken ghrelin-26-C8), synthesized at Daiichi Suntory Pharma Co. Ltd., Institute for Medicinal Research and Development (Gunma, Japan), was used as the standard peptide instead of rat ghrelin. ^125^I-(Tyr29)- rat ghrelin was used as a tracer (15,000 cpm per tube). All samples were run in one unique assay and the intra-assay coefficients of variation was 5.4%; the detection limit of the assay was 0.5 fmol per tube. Due to insufficient plasma volumes, we lack corticosterone and ghrelin measurements in two samples (corticosterone: two males of each sampling phase, ghrelin: 2 non-migratory females).

### 2.5 Data analyses

Statistical analyses were performed in R v3.6.2 (66) integrated in RStudio v1.3.1093 (67). We first assessed the association between plasma ghrelin and plasma corticosterone using Pearson’s tests regardless of sampling group and separately by sampling group. We then performed separate General Linear Models (GLMs, built-in function in the R environment) to assess for differences in plasma ghrelin or plasma corticosterone concentrations in relation to the sampling group (migratory or non-migratory), sex and their interaction. Corticosterone data were log-transformed to improve normality of model residuals. As in the main study population the migratory birds were on average fatter, ingested more food, and were more active at night than non-migratory birds (**Figure 1**), in order to avoid covariation among these variables and sampling group we performed separate GLMs for each sampling group to assess for links between each hormone with respect to food intake and migratory restlessness. As body mass dynamics is an integrative tissue measure of migratory behavior (24), we also used similarly structured GLMs to test whether plasma ghrelin and plasma corticosterone predicted either the total amount of body mass gained until the birds reached their fuelling peak (i.e., body mass change between experimental day 0 and the day in which the birds allocated to the migratory group were singly housed, herein referred as “total body mass gained”) or the total amount of body mass depleted as they entered the non-migratory status (i.e., body mass change between experimental day 70 and the day in which birds allocated to the non-migratory group were singly housed – herein referred as “total body mass lost”). As both corticosterone and ghrelin can also induce short-term changes in fuel metabolism e.g., (68, 69), we performed two additional models to explore whether levels of plasma ghrelin and plasma corticosterone would reflect within-individual changes in body mass detected over the last two-four weeks preceding blood sampling (migratory birds: body mass gained between experimental day 42 and the day of single housing – herein referred as “recently gained body mass”; non-migratory birds: body mass lost between experimental day 91 and the day of single housing – herein referred as “recently lost body mass”). We note that there was no association between either plasma ghrelin or corticosterone and variability in sampling date within each sampling group (migratory: -0.31< Pearson’s r < 0.36, *p* ≥ 0.17, non-migratory: -0.15 < Pearson’s r < -0.02, *p* ≥ 0.59) and thus we did not enter sampling date in the GLMs. In all models, non-significant interactions (*p* > 0.05) were sequentially dropped from the final models. All models met the assumptions of normality and homogeneity, which were assessed *via* graphical diagnostics of the residuals (70). Unless otherwise specified, descriptive statistics are provided as means ± se.

## 3 Results

**Figure 2:**
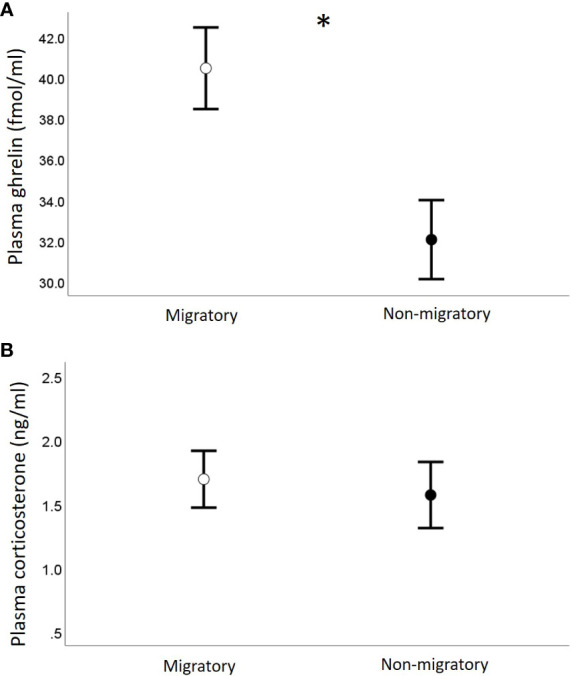
Concentrations of plasma ghrelin **(A)** and plasma corticosterone **(B)** in relation to sampling phase. Data are shown as mean ± se; * indicates *p* < 0.05.

**Figure 3:**
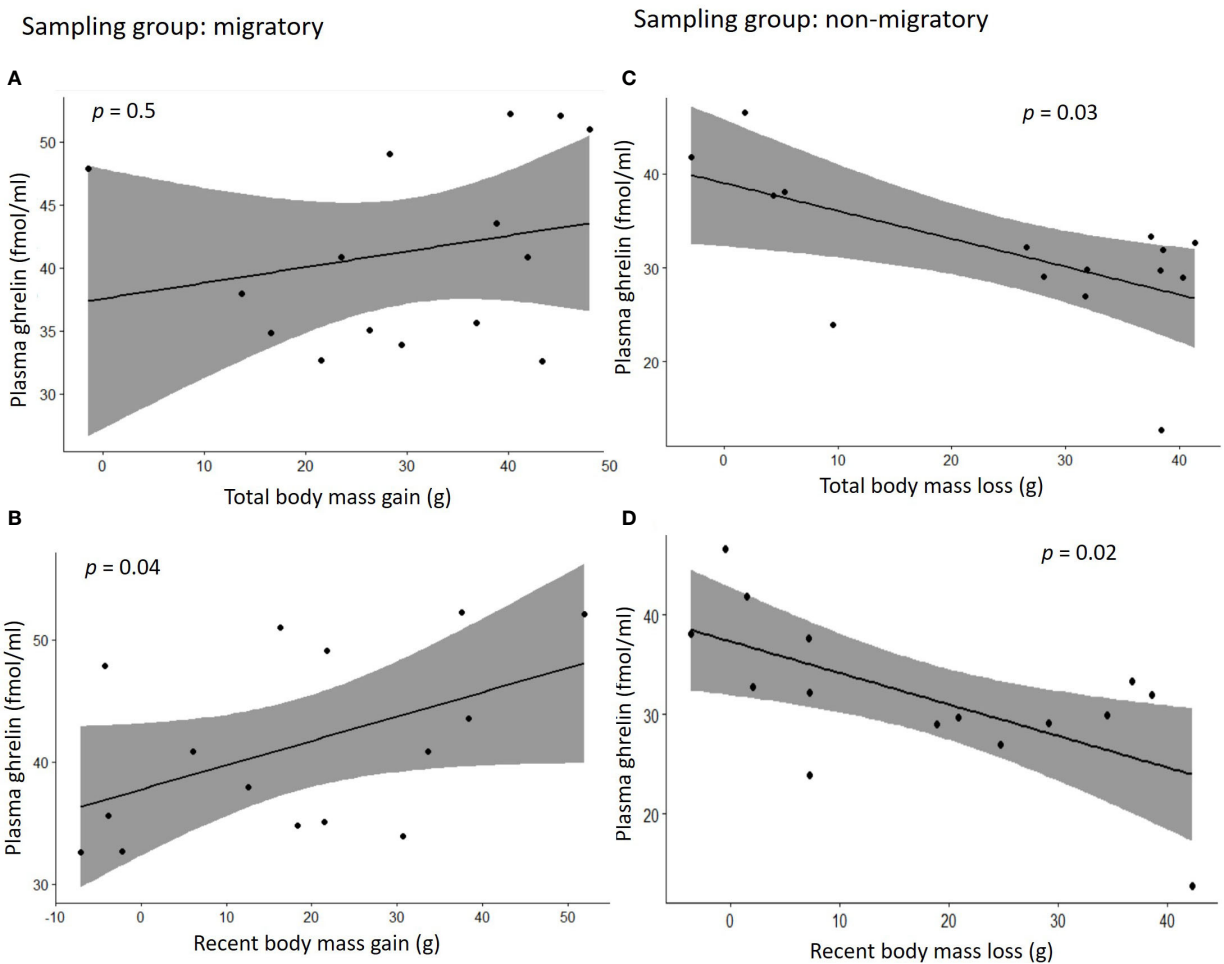
Correlation plots between concentrations of plasma ghrelin and changes in body mass during the migratory phase **(A, B)** or the non-migratory phase **(C, D)**. In **(A)** the total body mass gain was calculated as the difference in body mass between the time in which migratory birds were singly housed for blood sampling (experimental days 56-70) and experimental day 0; in **(B)** the recent body mass gain was calculated as the difference in body mass between the day of single housing and experimental day 42; in **(C)** the total body mass loss is the difference between the time in which non-migratory birds were singly housed for blood sampling (experimental days 105-119) and experimental day 70, and in **(D)** the recent body mass loss was calculated as the difference in body mass between the day of single housing and experimental day 91. Grey area in the graphs represents 95% confidence interval.

We found no correlation between plasma acylated ghrelin levels and plasma corticosterone (Pearson r = -0.12, *p =* 0.5; separately by sampling group: Pearson r > -0.11, *p* > 0.3). Migratory quails of both sexes had remarkably higher plasma ghrelin concentrations compared to quails sampled during the non-migratory phase (*p* = 0.004, **Table 1A** and **Figure 2** **A**), while we found no effect of sampling phase nor sex on plasma corticosterone (**Table 1B**; **Figure 2** **B**). Within each sampling group, we found no association of plasma ghrelin or plasma corticosterone with either food intake or migratory restlessness (**Tables 2A, B**). In the migratory sampling phase, the total amount of body mass gained was not associated with either plasma corticosterone or plasma ghrelin (**Table 2A** and **Figure 3** **A**), though plasma ghrelin but not plasma corticosterone did correlate with the recently gained body mass (**Table 2A** and **Figure 3** **B**). In the non-migratory sampling phase, the total amount of body mass lost was negatively associated with plasma ghrelin (*p* = 0.03, **Table 2B** and **Figure 3** **C**), but not with plasma corticosterone (**Table 2B**). Similarly, lower levels of plasma ghrelin also reflected larger recent losses in body mass (*p* = 0.02, **Table 2B** and **Figure 3** **D**).

**Table 1:** Results of GLMs assessing the effects of sampling phase and sex on plasma concentrations of (a) ghrelin or (b) corticosterone.

(a) Plasma ghrelin				
	Estimate	SE	t	p
Intercept	0.49	0.19	2.57	0.02
**Phase (non-migratory)**	**-8.40**	**2.70**	**-3.11**	**0.004**
Sex (male)	-4.55	2.73	-1.67	0.11
Phase: Sex*				0.98
**(b) Plasma corticosterone**				
	**Estimate**	**SE**	**t**	**p**
Intercept	0.49	0.19	2.57	0.02
Phase (non-migratory)	-0.16	0.21	-0.76	0.45
Sex (male)	-0.17	0.21	-0.79	0.44
Phase: Sex*				0.80

Factor estimates are indicated in parentheses. Significant factors (*p* < 0.05) are in bold; * indicates non-significant interaction term removed from the final models.

**Table 2:** Results of GLMs, separately by sampling group, assessing the effects of plasma corticosterone, plasma ghrelin and sex on migratory status assessed through changes in body mass, levels of food intake and migratory restlessness (for full details on study design see Material and Methods).

(a) Migratory birds				
*Total body mass gain*				
	Estimate	SE	t	p
Intercept	15.00	27.22	0.55	0.59
Corticosterone	0.94	4.73	0.20	0.85
Ghrelin	0.38	0.56	0.69	0.51
Sex (male)	-4.28	8.04	-0.53	0.61
***Recent body mass gain***				
	**Estimate**	**SE**	**t**	**p**
Intercept	-55.96	29.70	-1.88	0.09
Corticosterone	6.21	5.16	1.20	0.25
**Ghrelin**	**1.41**	**0.61**	**2.33**	**0.04**
Sex (male)	9.91	8.77	1.13	0.28
***Food intake***				
	**Estimate**	**SE**	**t**	**p**
Intercept	0.11	6.75	0.02	0.99
Corticosterone	0.46	1.17	0.39	0.70
Ghrelin	0.14	0.14	1.02	0.33
Sex (male)	1.56	1.99	0.78	0.45
***Migratory restlessness***				
	**Estimate**	**SE**	**t**	**p**
Intercept	0.12	0.12	1.01	0.33
Corticosterone	-0.02	0.02	-0.88	0.40
Ghrelin	0.002	0.002	0.75	0.47
Sex (male)	-0.01	0.03	-0.20	0.85
(b) Non-migratory birds				
*Total body mass loss*				
	**Estimate**	**SE**	**t**	**p**
Intercept	-64.78	18.68	3.47	0.01
Corticosterone	-4.50	3.27	-1.38	1.38
**Ghrelin**	**-1.20**	**0.47**	**-2.54**	**0.03**
Sex (male)	-9.18	6.98	-1.32	0.22
***Recent body mass lost***				
	**Estimate**	**SE**	**t**	**p**
Intercept	62.43	19.89	3.14	0.01
Corticosterone	-2.63	3.48	-0.76	0.47
**Ghrelin**	**-1.32**	**0.50**	**-2.62**	**0.02**
Sex (male)	2.15	7.43	0.29	0.78
***Food intake***				
	**Estimate**	**SE**	**t**	**p**
Intercept	7.18	3.90	1.84	0.09
Corticosterone	-1.40	0.68	-2.05	0.07
Ghrelin	0.02	0.10	-2.05	0.82
Sex (male)	-0.01	1.46	-0.01	0.99
(b) Non-migratory birds				
*Migratory restlessness*				
	**Estimate**	**SE**	**t**	**p**
Intercept	0.05	0.20	0.25	0.81
Corticosterone	-0.02	0.03	-0.54	0.60
Ghrelin	0.01	0.01	1.01	0.33
Sex (male)	0.08	0.07	1.07	0.31

Fixed factor estimates are indicated in parentheses; significant terms (*p* < 0.05) are in bold.

## 4 Discussion

In this study, we exposed Common quails to controlled changes in day length to simulate autumn migration followed by a non-migratory wintering phase. We compared plasma corticosterone and ghrelin concentrations between the two sampling phases and assessed whether these two metabolic hormones vary between migratory states. In accordance with our predictions, we found that the emergence of the migratory phenotype was associated with higher levels of plasma ghrelin and that plasma ghrelin was associated with changes in body mass as birds transitioned into the autumnal migratory state, or the wintering non-migratory state. Contrary to our predictions, we observed no correlation between ghrelin and circulating levels of the metabolic hormone corticosterone, which also did not differ between migratory and non-migratory birds and was not associated with changes in body mass, food intake or migratory restlessness that accompanied the switching of the migratory phenotype.

This study allowed us to cross check function of ghrelin known for domestic Galliformes with the recent findings on few migratory Passerines by studying a migratory Galliform in controlled laboratory conditions. The elevation of plasma ghrelin in the fattened migratory quails relative to the leaner non-migratory quails we found is in line with the garden warbler study by Goymann et al. (49) showing a positive relationship between plasma ghrelin and fat stores in spring migrating birds caught at a stopover site. On the other hand, our results are in contrast with the study by Eikenaar et al. (51) in common blackbirds (*Turdus merula*) in which such association was not found in birds sampled at a stopover during autumn migration. However, it is difficult to draw a direct comparison between the previous studies on ghrelin in migratory birds, which were all performed in actively migrating birds, and ours that instead involved long-term housing of birds and manipulation of photoperiod conditions to induce the transitioning of the migratory state. Regardless, an important caveat of our study design is that the nutritional and migratory state of the birds were inter-connected given that all migratory birds were sampled at a standardised time, when they reached their peak in body mass/fat stores. We cannot, therefore, rule out the possibility that plasma ghrelin might simply reflect the birds’ nutritional conditions without necessarily having a role in the mediation of migratory behaviour. However, this possibility appears somewhat unlikely given that we also found associations between plasma ghrelin and changes in body mass within the two sampling phases. Specifically, while plasma ghrelin did not covary with the total amount of body mass gained since the beginning until completion of the fattening process, it did positively covary with the body mass gained over its intermediate/final stages (i.e., recent body mass gain) in partially fattened birds. Despite this finding is based on a correlative approach, it raises the intriguing possibility that elevated levels of ghrelin might contribute to inhibit further pre-migratory fuelling as the injection of chicken ghrelin induces short-term anorectic effects by stimulating the breakdown of lipids (46, 64) and rapidly stimulates migratory movements in the wild (50). This possibility is further supported by the associations we found between ghrelin and the rapid depletion in body mass observed within the birds sampled during the non-migratory phase, when we observed lower levels of ghrelin now reflecting larger losses in body mass both over the long- and short-time frame (i.e., over an average of six or three weeks preceding blood sampling, respectively). These results suggest that ghrelin may regulate body mass *via* a negative feedback loop with high circulating concentrations of ghrelin exerting inhibitory effects on further increases in fattening. Nevertheless, the exact mechanisms underlying the regulation of food intake and avian migratory fattening by the ghrelin axis remain to be fully elucidated. The lack of correlation of plasma ghrelin with both food intake and levels of migratory restlessness that we assessed in close proximity of blood sampling (1-3 days earlier) could be interpreted as contrasting with the latter hypothesis. However, this apparent contrast could be due to limited variance within the two sampling groups as all migratory birds were sampled at their peak of body mass and when migratory restlessness levels were high, while all non-migratory birds were sampled when most of the accumulated fuel reserves had been depleted and when migratory restlessness levels were low. In future work, a repeated sampling design within the same individual birds will be one among the key necessary steps to experimentally verify whether ghrelin has a functional/causative role in mediating fuelling dynamics typical of phenotypic transitions between migratory states.

Contrary to our prediction, circulating levels of corticosterone were not elevated in migratory birds compared to non-migratory birds. This prediction was mostly based on previous work in captive birds reporting a relatively large up-regulation in corticosterone secretion, especially detected in migrants that had reached stable and high levels of body mass at completion of pre-migratory fuelling (24, 26, 29, 30, 34). It is possible that the variance due to inter-individual variation in baseline levels of corticosterone (71) has masked the emergence of potential differences in corticosterone between migratory and non-migratory quails. However, we did not observe in our data a large variance in corticosterone levels, which were always very low and in a range comparable with baseline values reported for the closely related Japanese quail e.g., (72, 73). We also did not find associations between corticosterone levels and variability in sampling date within each sampling group. The neat study by Piersma et al. (30) in the Red knots demonstrated a rapid decline in plasma levels of corticosterone as soon as the fattened migrants started depleting body mass over the first seven days preceding blood sampling. As we performed blood sampling in the migratory birds over two consecutive weeks (experimental days 56-70), it could be argued that the depletion in energy reserves had already started in some birds, thus reducing our statistical power to detect an up-regulation in corticosterone secretion. However, we can exclude this potential confounding factor because body mass remained high, or even slightly increased, during this time-frame, and the signs of body mass depletion were only observed after experimental day 70. Thus, in our study species it seems unlikely that corticosterone up-regulation (at least of the magnitude reported in previous work) contribute in sustaining high levels of pre-migratory fuelling. On the other hand, in accordance with many previous studies [reviewed in (24); e.g (30, 32, 74, 75)], we found that circulating levels of corticosterone did not reflect amounts of body mass gained or depleted before blood sampling as birds transitioned to the autumnal migratory state or to the wintering non-migratory state, respectively. These results provide further support to the idea that corticosterone has a very limited, if any, role in stimulating physiological preparations for migration (24). Corticosterone could, however, have a role in promoting or sustaining migratory departure as studies conducted in the wild suggested (76–79). Here, we did not find a correlation between plasma corticosterone and migratory restlessness, despite the latter being higher in the migratory birds. This apparent discrepancy could be due to several aspects of study design associated to our temporal sampling scale. Importantly, we did not perform a long-term tracking of activity levels over the transitioning phases and we did not take corticosterone measurements during or just before the hours in which migratory restlessness is actually expressed (i.e., afternoon, or night given that quails are nocturnal migrants (54) when corticosterone levels would be predicted to be higher.

We did not find a relationship between circulating levels of plasma ghrelin and plasma corticosterone. We predicted such a relationship to be positive because the peripheral administration of ghrelin induces corticosterone release in the closely related chickens (46–48). Despite plasma ghrelin was relatively high in the migratory birds, it is possible that such increase was not large enough to induce a positive feedback on the Hypothalamic-Pituitary-Adrenal axis (HPA axis) and further manipulative experiments would be needed to clarify if ghrelin has glucocorticoid stimulatory effects. We cannot exclude the possibility that a potential relationship between ghrelin and corticosterone was missed due to a lag in the response time, as it would happen if peaks in ghrelin and corticosterone secretion occur at different times respect to changes in body mass associated to different migratory states. Again, a repeated-measure design experiment would be extremely useful to test this possibility. Nevertheless, our results are consistent with the only previous study that tested a functional relationship between ghrelin and corticosterone in a migratory bird, which reported no correlation between plasma levels of the two hormones in migrants close to depart at stopover (51). Further studies will be needed to clarify whether there is (or not) a functional interplay between ghrelin and corticosterone in relation to modulation of migratory behaviour.

## Data availability statement

The original contributions presented in the study are included in the article/**Supplementary Material**. Further inquiries can be directed to the corresponding author.

## Ethics statement

The animal study was reviewed and approved by Ethics Committee of the University of Veterinary Medicine Vienna, and the Federal Ministry of Science, Research and Economy (BMWFW-68.205/0037-WF/V/3b/2017).

## Author contributions

Conceptualization: VM and LF; Methodology: VM, HK, GP, and LF; Formal analysis: VM; Resources: VM, HK, and LF; Writing – original draft: VM; Writing – review & editing: VM, HK, and LF; Project administration: VM; Funding acquisition: VM and LF. All authors contributed to the article and approved the submitted version.
