# Supplementary material for: Ghrelin, not corticosterone, is associated with transitioning of phenotypic states in a migratory Galliform
Source: Front Endocrinol (Lausanne). 2023 Jan 9;13:1058298. doi: 10.3389/fendo.2022.1058298 (PMC9869107; doi:10.3389/fendo.2022.1058298)
Supplement: Supplementary file 1 [file DataSheet_1.pdf]

Supplementary Material

1 Supplementary Material and Methods

1.1 Assessment of food intake and migratory restlessness at sampling

For every individual bird, we measured daily food consumption upon the start of the single housing (10:00–11:00 h) until the following 24 h by weighing the remaining food in the bowl and any food pellet that had been scattered around the cage by the bird. Food intake was calculated as the difference between the mass of the remaining food from the mass of the food provided at the beginning of the experiment. In a subset of birds (n=12) food intake was measured for two consecutive nights and values were highly repeatable within each individual (r=0.93, p <0.0001, (1)). For every bird, we also continuously monitored nocturnal locomotor activity during the first two nights since the start of the single housing. Each cage was equipped with an infrared sensor connected with an activity recorder that registered locomotor activity within the cage in 1-min intervals. For each night, we calculated the average activity levels for each bird. Average activity values between the two consecutive night were highly correlated (Pearson's test, r = 0.85, p < 0.0001) and measurement repeatability within each individual bird was very high (r=0.90, p < 0.0001, (1)). Thus, for the further statistical analyses (paragraph below) we used the average activity levels during the second night of single housing (i.e. migratory restlessness).

1.2 Data Analyses

Two separate General Linear Mixed Models (GLMMs) were performed to assess the effects of the photoperiod manipulation changes in body mass and fat scores. The first model used the data collected from experimental days 0–56 (i.e. period of weekly reduction in day length); the second model used the data collected from experimental days 70–105 (i.e. when the photoperiod remained constant). For both analyses, we entered time, sex, and their interaction as fixed factor and individual bird identity as random factor to control for the presence of repeated measurements. We used separate General Linear Models (GLMs) to assess whether food intake or migratory restlessness differed in relation to sampling phase (migratory versus non-migratory), sex and its interaction to check for sex-specific differences in food intake between the two sampling phases. Non-significant interaction terms were removed from the main model, while when significant they were further explored by performing simple slopes analyses (R package "interactions"- (2)).

Table S1. Results of generalised linear mixed models (GLMMs) with a Gaussian distribution error to assess the effects of the photoperiod manipulation on body mass and subcutaneous fat scores performed in a captive population of common quail in order to simulate autumn migration followed by a non-migratory life-history stage. Fixed factors estimates are indicated in parenthesis, r indicates random factor and its associated variance, in bold significant terms ($p < 0.05$). Details were reported in detail elsewhere (3). Sample size for analyses shown in (a) and (b): n = 68; sample size for analyses shown in (c) and (d): n = 35.

	(a) Body mass experimental days 0-56					(b) Fat scores experimental days 0-56				
	**Estimate**	**SE**	**df**	**t**	***p***	**Estimate**	**SE**	**df**	**t**	***p***
Bird id (r)	100.410					4.291				
Residual	95.750					10.403				
Intercept	104.474	2.155	112.242	48.485	<0.0001	4.282	0.557	163.320	7.694	<0.0001
**Day**	**0.369**	**0.038**	**270.000**	**9.727**	**<0.0001**	**0.103**	**0.012**	**270.000**	**8.256**	**<0.0001**
**Sex (Male)**	**-6.449**	**3.047**	**112.242**	**-2.116**	**0.037**	-0.488	0.787	163.320	-0.620	0.536
Time: Sex	0.035	0.054	270.000	0.662	0.509	0.007	0.018	270.000	0.369	0.713

	(c) Body mass experimental days 70-105					(d) Fat scores experimental days 70-105				
	**Estimate**	**SE**	**df**	**t**	***p***	**Estimate**	**SE**	**df**	**t**	***p***
Bird id (r)	184.580					10.955				
Residual	50.410					4.218				
Intercept	153.888	6.294	166.397	24.450	<0.0001	17.413	1.755	170.917	9.924	<0.0001
**Day**	**-0.302**	**0.060**	**138.000**	**-4.993**	**<0.0001**	**-0.072**	**0.018**	**138.000**	**-4.117**	**<0.0001**
**Sex (Male)**	**29.043**	**9.309**	**166.397**	**3.120**	**0.002**	**5.393**	**2.595**	**170.917**	**2.078**	**0.039**
**Time: Sex**	**-0.420**	**0.089**	**138.000**	**-4.699**	**<0.0001**	**-0.071**	**0.026**	**138.000**	**-2.755**	**0.007**

Slope analysis for significant interaction terms in (c) and (d): the decrease in body mass and fat stores was stronger males compared to female quails (body mass - male: -0.72 ± 0.07, t = -10.96, $p < 0.001$; female: -0.30 ± 0.06, t = -4.99, $p < 0.001$; fat scores - male: -0.14 ± 0.02, t = -7.52, $p < 0.001$, female: -0.07 ± 0.02, t = -4.12, $p < 0.001$).

Table S2. Results of General Linear models (GLM) assessing whether (a) food intake or (b) migratory restl1essness differed in relation to sampling group, sex, and their interaction. Fixed factors estimates are indicated in parenthesis, in bold significant terms ($p < 0.05$), *non-significant interaction term was removed from the final model.

(a) Food intake

	**Estimate**	**SE**	**t**	***p***
Intercept	7.720	0.714	10.818	<0.0001
**Group (Non-Migratory)**	**-2.125**	**0.794**	**-2.676**	**0.009**
Sex (Male)	-0.364	0.794	-0.459	0.648
Group: Sex*				1.00

(b) Migratory restlessness

	**Estimate**	**SE**	**t**	***p***
Intercept	0.25035	0.0501	4.997	<0.0001
**Group (Non-Migratory)**	**-0.11392**	**0.05573**	**-2.044**	**0.045**
**Sex (Male)**	**0.12513**	**0.05571**	**2.246**	**0.0281**
Group: Sex*				0.08

2 Supplementary References

1. Lessells CM, Boag PT. Unrepeatable Repeatabilities: A Common Mistake. *Auk* (1987) 104(1):116-21. doi: 10.2307/4087240.

2. Long JA. Interactions: Comprehensive, User-Friendly Toolkit for Probing Interactions (2019). Available from: https://CRAN.R-project.org/package=interactions

3. Marasco V, Sebastiano M, Costantini D, Pola G, Fusani L. Controlled Expression of the Migratory Phenotype Affects Oxidative Status in Birds. *J Exp Biol* (2021) 224(6):jeb233486. doi: 10.1242/jeb.233486.
